# Editorial: Emerging roles and mechanisms of stromal cells in carcinomas at the molecular level

**DOI:** 10.3389/fimmu.2022.1025838

**Published:** 2022-09-08

**Authors:** Jaewoo Hong, Jun-O Jin, Wei-Yu Chen, Alessandro Poggi, Jae-Ho Cheong

**Affiliations:** ^1^ Department of Physiology, Daegu Catholic University School of Medicine, Daegu, South Korea; ^2^ Department of Microbiology, University of Ulsan College of Medicine, Seoul, South Korea; ^3^ Department of Biochemistry and Molecular Biology, College of Medicine, National Cheng Kung University, Tainan, Taiwan; ^4^ Molecular Oncology and Angiogenesis Unit, IRCCS Ospedale Policlinico San Martino, Genoa, Italy; ^5^ Department of Biochemistry and Molecular Biology, Brain Korea 21 PLUS Project for Medical Sciences, Yonsei University College of Medicine, Seoul, South Korea; ^6^ Department of Surgery, Yonsei University College of Medicine, Seoul, South Korea

**Keywords:** tumor microenvironment, stromal cell, carcinoma, cancer-associated fibroblast, solid tumor, immune surveillance, oral cancer

For an extended period, the tumor microenvironment (TME) has not been focused on in the field of cancer biology before Stephen Paget’s “seed and soil” hypothesis (1). The representative characters of cells involved in TME are high plasticity and continuous phenotypic and functional change. For example, the desmoplastic reaction in pancreatic cancer is a critical histological observation, tightly associated with significantly increasing the interstitial fluid pressure within the tumor niche. Furthermore, the desmoplastic stroma and compressed vessel delay or block the circulating therapeutic agents’ target location. Inflammation is well-known as a critical factor in developing TME enhancing tumorigenesis and cancer promotions in carcinomas.

Stromal and immune cells usually surround and harmonize with cancer cells or mass, forming the inflammatory TME. Interactions between tumor cells and tumor-associated stromal cells (TASCs) have critical roles in tumor growth and progression. In this context, among stromal cells, fibroblast-like cells, mesenchymal stromal cells, and carcinoma-associated fibroblasts can be considered the main players involved in either pro- or anti-tumorigenic effects. The complexity of stroma-tumor interaction shows remarkable heterogeneous tumor mass formation even though this process has high similarity with normal wound healing processes such as neoangiogenesis, fibroblast, and immune cell infiltration.

Ten articles were contributed to this specific Research Topic and classified into the following categories: three original research (Gao et al., Joo et al., and Peng et al.) and six reviews (Hwang et al., Tagirasa and Yoo, Kim et al., Shim et al., Mun et al., Kim et al., and Koppensteiner et al.).

Gao et al. revealed the upregulation of PD-L1 expression in colorectal cancer (CRC) by cancer-associated fibroblasts (CAFs) is mediated by Akt phosphorylation. Since CAFs are one of the major components of TME and exert as immune regulators to generate immune suppression in TME. In this study, the upregulation of PD-L1 expression in CRC by CAFs through the activation of Akt was confirmed with colorectal cancer cell lines and also with human CRC patients in correlation with the disease-free survival. Koppensteiner et al. reviewed the immune regulating effects of CAFs in anti-cancer T cell therapy. In this review, the authors suggested the interplay of T cells and CAFs by bidirectional crosstalk plays a significant role in TME. They discussed various mechanisms by the interplay and crosstalk of CAFs and T cells that leads to the negative anti-cancer immune responses.

We have two review articles addressing specific molecular families affecting TME originating from stromal environments. First, Tagirasa and Yoo reviewed an exciting point of view on the tumor-stroma interface. They focused on the enzymatic activities of serine proteases, while most other contributors focused more on cellular effects on TME. They dealt with stromal serine proteases such as fibroblast-activation protein, urokinase-type plasminogen activator, kallikrein-related peptidases, and granzymes that led to the tumor progression and discussed the therapeutic applications. Secondly, Shim et al. discussed the IL-32 subfamily affecting TME. Each IL-32 subtype has a different role on cancer cells, and the stromal IL-32 is still unclear to the TME yet, but they discuss IL-32 as a possible regulatory role in cancers.

Two original articles revealed the novel mechanisms of TME affected by the stromal environments. Joo et al. told a new subset of CAF using single-cell RNA sequencing from frozen skin tissue with adult T-cell leukemia/lymphoma (ATLL) patients. Their study identified a novel CAF subset with enhanced EGR1 and EGR2 expression. These cells can highly proliferate CD4 T cells *via* FGF7-FGF1 and PDGFA-PDGFRA/B signaling. They are also associated with the CD8 and NKT subset expansions, which can be a new therapeutic target in the future. Peng et al. state the evasion of NK cell immune surveillance *via* cytoskeleton remodeling. They discovered cancer cells resistant to the immune responses through enhanced vimentin and actin reorganization. This was also observed from human tumor samples, which may have clinical value in terms of cancer diagnostics.

Kim et al. discussed the role of stroma in specific cancer subsets such as an endometrial tumor. They were focusing on stromal tumors rather than carcinomas. Endometrial stromal tumor is a rare subset of cancer, and they categorized the disease categories according to the genetic alteration and suggested possible therapeutic approaches. Indeed, the discovery of drugs to treat stromal tumor may represent a powerful approach to affect the pro-tumorigenic effect of stromal cells in carcinomas.

Mun et al. reviewed the interplay of immune cells and stromal cells in the tumor microenvironment. They discussed about the positive and negative relationships from the point of view of tumor development for use in research applications and therapeutic strategies. Kim et al. reviewed the solid tumor and TME. They discuss the adaptation process of tumors to adverse environments *via* communication with neighboring cells to overcome unwanted growth conditions.

This Research Topic focuses on enlightening the current and recent findings on the interplay between cancer inflammation and TME to understand the obstacles of cancer therapy for epithelial cancers. Cancer immunotherapy is one of the powerful strategies to cure cancer since engineered cell therapy has been popular recently, such as oncolytic viruses, antibody therapies, and CAR-T therapy (2). However, most current immunotherapies have limitations in targeting solid tumors like carcinomas. Tumor microenvironment-targeting therapy for carcinomas has the unmeasurable potential to synergize the current immunotherapy if we overcome the current hurdles (3). The etiologies of immunotherapy resistance are multi-layered, not only the issue of tumor cells but also the complexity of the interplay between carcinomas and the microenvironment in their solid mass. One reason to look at the stromal cells in carcinomas in the aspect of inflammation is that TASCs secrete many inflammation-related molecules, including IL-6, IL-8, stromal-derived factor-1 alpha VEGF, besides TGFβ. These molecules can trigger carcinoma cell growth and subsequent metastasis through epithelial-mesenchymal transition improving the pro-tumorigenic properties of TME. Solving the puzzles of tumor microenvironment and inflammation in carcinomas at the molecular levels will enable us to address currently unsolved problems in understanding malignant carcinomas’ mechanisms and therapeutic directions.

## Author contributions

All authors listed have made a substantial, direct, and intellectual contribution to the work and approved it for publication.

## Funding

This work was supported by the grant of Research Institute of Medical Science, Daegu Catholic University (2022) to JH.

## Acknowledgments

We would like to thank all the authors who have participated in this Research Topic and the reviewers for their invaluable comments.

## Conflict of interest

The authors declare that the research was conducted in the absence of any commercial or financial relationships that could be construed as a potential conflict of interest.

## Publisher’s note

All claims expressed in this article are solely those of the authors and do not necessarily represent those of their affiliated organizations, or those of the publisher, the editors and the reviewers. Any product that may be evaluated in this article, or claim that may be made by its manufacturer, is not guaranteed or endorsed by the publisher.
